# Epigenetic analysis in a murine genetic model of Gulf War illness

**DOI:** 10.3389/ftox.2023.1162749

**Published:** 2023-06-14

**Authors:** Khyobeni Mozhui, James P. O’Callaghan, David G. Ashbrook, Pjotr Prins, Wenyuan Zhao, Lu Lu, Byron C. Jones

**Affiliations:** ^1^ Department of Preventive Medicine, College of Medicine, University of Tennessee Health Science Center, Memphis, TN, United States; ^2^ Department of Genetics, Genomics and Informatics, College of Medicine, University of Tennessee Health Science Center, Memphis, TN, United States; ^3^ Molecular Neurotoxicology Laboratory, Toxicology, and Molecular Biology Branch, Health Effects Laboratory Division, U. S. Centers for Disease Control and Prevention, NIOSH, Morgantown, WV, United States; ^4^ Department of Pharmacology, Addiction Science, and Toxicology, College of Medicine, University of Tennessee Health Science Center, Memphis, TN, United States

**Keywords:** MBD-seq, DNA methylation, diisopropyl fluorophosphate, corticosterone, systems genetics

## Abstract

Of the nearly 1 million military personnel who participated in the 1990–1991 Gulf War, between 25% and 35% became ill with what now is referred to as Gulf War Illness (GWI) by the Department of Defense. Symptoms varied from gastrointestinal distress to lethargy, memory loss, inability to concentrate, depression, respiratory, and reproductive problems. The symptoms have persisted for 30 years in those afflicted but the basis of the illness remains largely unknown. Nerve agents and other chemical exposures in the war zone have been implicated but the long-term effects of these acute exposures have left few if any identifiable signatures. The major aim of this study is to elucidate the possible genomic basis for the persistence of symptoms, especially of the neurological and behavioral effects. To address this, we performed a whole genome epigenetic analysis of the proposed cause of GWI, viz., exposure to organophosphate neurotoxicants combined with high circulating glucocorticoids in two inbred mouse strains, C57BL/6J and DBA/2J. The animals received corticosterone in their drinking water for 7 days followed by injection of diisopropylfluorophosphate, a nerve agent surrogate. Six weeks after DFP injection, the animals were euthanized and medial prefrontal cortex harvested for genome-wide DNA methylation analysis using high-throughput sequencing. We observed 67 differentially methylated genes, notably among them, *Ttll7, Akr1c14, Slc44a4,* and *Rusc2,* all related to different symptoms of GWI. Our results support proof of principle of genetic differences in the chronic effects of GWI-related exposures and may reveal why the disease has persisted in many of the now aging Gulf War veterans.

## Introduction

In 1991, the United States deployed 700,000 military personnel to Kuwait in response to Saddam Hussein’s invasion of that country. An additional 200,000 plus were deployed from the United States allies. During the conflict, between 25% and 35% of the military personnel complained of a multisymptom malaise now generally referred to as Gulf War Illness (GWI; David et al., 1997). Reported symptoms include gastrointestinal distress, pain, neurological complaints (Duong et al., 2022) and sickness-related behaviors including lethargy, “brain fog,” and a general inability to function effectively (Engdahl et al., 2018). Remarkably, among most of those afflicted, the complaints persist to this day, more than 30 years since the end of the conflict. The underlying basis of the malady is open to debate; however, there is evidence that many of the troops were exposed to organophosphates, either as insecticides or nerve agents due to the destruction of an ammunition dump that inadvertently released sarin and cyclosarin with the potential exposure of up to 100,000 military personnel (Chao et al., 2011). Both clinical and preclinical research now indicate that the neurological and behavioral effects seen in GWI were the result of neuroinflammation produced by exposure to organophosphates (OP) coupled with increased levels of circulating glucocorticoid hormones, i.e., cortisol, as might be expected due to the high physiological stress of the war theater (Yates et al., 2021). Indeed, when subjected to testing in an animal model, the major rodent glucocorticoid hormone, corticosterone (CORT), was shown to greatly increase the expression of proinflammatory cytokine genes in C57BL/6J (B6) mice (Locker et al., 2017).

The B6 strain is often contrasted with the DBA/2J (D2) strain as there are marked differences in stress response and sensitivity between the two strains (Matthews et al., 2008; Mozhui et al., 2010; Terenina et al., 2019). B6 and D2 are also the parent strains for the BXD family, a large panel of genetically diverse recombinant inbred and advanced intercross strains that are a mosaic of the B6 and D2 genomes (Ashbrook et al., 2021) The genomes of the B6 and D2 strains have been fully sequenced, and there are over 6 million genetic variants that differ between the two (Ashbrook et al., 2021). Furthermore, in our previous work, we have shown that treatment with CORT and OP results in genetically driven differences in gene expression in the BXDs (Xu et al., 2020). The fact that only 25%–30% of Gulf War veterans developed GWI indicates a genetic component, and the contrast between B6 and D2 provides a powerful model system to test the interaction between environmental exposures and genetics. As our work from 2017 (Locker et al., 2017) demonstrated that B6 has high sensitivity to the combination of CORT + OP, we conducted a pilot study to compare between the two strains, and between sexes. Indeed, in our unpublished pilot study, we found that D2 mice and females of both strains were much less sensitive to the pro-inflammatory effects of CORT + OP. Follow-up research in 30 recombinant inbred strains derived from B6 and D2 intercrosses led to the nomination of two genes that underlie individual differences in susceptibility to neuroinflammation produced by combined exposures (Jones et al., 2020; Gao et al., 2020).

Now that we have demonstrated a possible genetic basis for individual differences in susceptibility to exposures that underlie GWI in the mouse model, the major question remaining is why most of those so afflicted have been sick for so long. The persistence following the initial exposure indicates long-term changes in cellular properties that may involve epigenetic remodeling (Bielawski et al., 2019). DNA methylation is particularly relevant in this context as CpGs are a crucial interface between the genome and the environment. CpG methylation is a canonical epigenetic modification that entails adding a methyl group to a CG dinucleotide, and this epigenetic mechanism plays a critical role in maintaining stable gene expression (Illingworth and Bird, 2009). Consistent with the potential role of the methylome in GWI, studies in humans have shown the GWI is associated with significant differences in DNA methylation of CpGs (Trivedi et al., 2019). Several studies have also shown that exposure to environmental toxins as well as stressors can results in extensive alteration to CpG methylation, and this could be a mechanism by which these exposures result in long-term changes and illness (Houtepen et al., 2016; van der Plaat et al., 2018).

Given that B6 and D2 have genetic and gene expression differences in their reponse to CORT + OP treatment, our goal is to examine the methylome as a potential basis for gene-by-environment interaction. To that end, here we report the outcome of our study that subjected B6 and D2 strains to an organophosphate compound coupled with high circulating glucocorticoid hormone (corticosterone) followed by genome-wide profiling of DNA methylation in the prefrontal cortex using the affinity-based method, MBD-seq (Aberg et al., 2018; Aberg et al., 2020; Sandoval-Sierra et al., 2020). Our main goal is to demonstrate genetic differences in the epigenetic effects of CORT + OP exposure as justification to launch a study in multiple strains of BXD recombinant inbred mouse strains.

## Materials and methods

### Mice and treatment

The animals used in this study were 23 male and female B6 and D2 mice, 140–190 days of age. Assignment by strain, sex, and treatment is presented in Table 1 (see below). Treatment consisted of corticosterone in the drinking water (20 mg% w/v) for 7 days followed by i.p. injection of 4.0 mg kg^−1^ diisopropyl fluorophosphate (DFP, an OP sarin surrogate). The animals were then treated with corticosterone in their drinking water every other week for 12 weeks after which, the animals were euthanized by cervical dislocation and the brain harvested and dissected to yield the prefrontal cortex. We refer to the treatment group as CORT + DFP. The control group followed the same schedule but withour conrticosterone in the drinking water and saline injection. All procedures involving animals were approved by the UTHSC Institutional Animal Care and Use Committee.

**TABLE 1:** Sample details. Age is in postnatal days.

	C57BL/6J (B6)				DBA/2J (D2)			
	Females		Males		Females		Males	
Treatment	n	Age ±SD	n	Age ±SD	n	Age ±SD	n	Age ±SD
CORT + DFP	3	169 ± 42	3	199 ± 47	3	163 ± 28	3	163 ± 28
Control	2	145 ± 0	3	145 ± 0	3	163 ± 28	3	179 ± 28

### Tissue harvest and DNA sample process

Genomic DNA was extracted from the prefrontal cortex (PFC) using the Quick-DNA/RNA Miniprep Plus kit (Zymo Research, Irvine, CA, United States) and checked for purity and quantity using a NanoDrop spectrophotometer (ThermoFisher Scientific, Waltham, MA, United States), and a Qubit™ fluorometer and the dsDNA BR (Broad Range) Assay kit (Invitrogen). Affinity-based CpG enrichment was done using the Invitrogen MethylMiner Methylated DNA Enrichment Kit (ThermoFisher Scientific, Waltham, MA, United States), which relies on the methyl-CpG binding domain protein 2 (MBD2) protein to capture DNA fragments containing methyl-CpGs. MBD2 preferentially binds to methylated CpGs, and this depletes the DNA sample of DNA regions without CpGs, and enriches for methyl-CpGs (Aberg et al., 2018; Aberg et al., 2020). First, 1 µg of DNA in 110 μL low TE (tris-EDTA) buffer was sheared to ∼150 bp fragments using a Covaris S2 ultrasonicator (Covaris, Woburn, MA, United States). Sonication settings were the same as described in Sandoval-Sierra (Sandoval-Sierra et al., 2020) with cycle/burst of 1 for 10 cycles of 60 s, duty cycle of 10%, and intensity of 5.0. DNA fragment size and quality were assessed using the Agilent Bioanalyzer 2100 (Agilent, Santa Clara, CA, United States). MBD-capture reaction was done according to the standard manufacturer’s protocol, followed by a single step elution with 2 M NaCl solution. The enriched DNA was then re-concentrated by ethanol precipitation, and the final concentration of methylated-CpG enriched DNA ranged from 0.17 to 2.1 ηg per μl (0.87 ± 0.39).

### Sequencing and initial data processing

Sequencing was performed to 40 million reads per sample (150 paired-end) on Illumina NovaSeq 6000 (Illumina, San Diego, CA, United States).

### Alignment to the reference genome

*Mus musculus* (mouse) reference genome (GRCm38) and gene model annotation files were downloaded from the Ensembl genome browser (https://useast.ensembl.org/). Indices of the reference genome were built using STAR v2.5.0a (Dobin et al., 2013) and paired-end clean reads aligned to the reference genome.

#### Quantification of DNA methylation

For quantification of DNA methylation, the bam files were loaded to the MEDIPS R package (version 1.44.0) (Lienhard et al., 2014) using the MEDIPS.createSet function with the following parameters: uniq = 1, extend = 150, ws = 150, shift = 0. This divided the mm10 mouse genome into 150 bp non-overlapping windows, and reads were counted for each bin. The MEDIPS.couplingVector function was used to compute the local CpG density (coupling factor or CF), and the read counts were normalized to the CF using the function MEDIPS.meth. To retain only 150 bp bins that had sufficient coverage for reliable quantification and statistical analyses, we implemented the following filters: 1) bins with no CpGs (CF = 0) and mean read counts ≤1 were excluded, resulting in 5,724,879 bins; 2) these were loaded to the EdgeR R package (version 3.34.1) (Robinson et al., 2010), and further filtered on the basis of counts per million (CPM) to retain only reads with more than 1 CPM in 2 or more libraries. This resulted in 210,191 CpG regions that were then normalized by the library size using the calcNormFactors function. RPKM values were then extracted using the parameters gene.length = 150, log = TRUE, and the CpG regions were annotated using the HOMER software (Heinz et al., 2010).

### Statistical analyses

Principal component analysis (PCA) using the full set of 210,191 CpG regions was performed on R using the prcomp() function. As no outliers were detected, all samples were included for downstream analyses. For epigenome-wide association analysis (EWAS), we applied the following regression model in R: CpG_i_ ∼ glm(treatment*strain + sex + age), where i is the CpG region from 1 to 210,191. To test whether the methylation differences induced by the DFP treatment were associated with changes in gene expression, we took RNA-seq data generated from the PFC of a panel of mice belonging to the BXD Family. These data have been described in detail (Xu et al., 2020). For the present work, we took the data from the untreated control cohort (n = 127 mice), and the corticosterone and DFP cohort (n = 129). For significant CpG regions identified by the EWAS, we linked the methylation sites to its cognate gene (nearest gene or gene in which the CpGs are located), and for this set, we performed linear regression with gene expression as the dependent variable and treatment as the main predictor with adjustment for sex.

## Results

### Data quality check and overall methylation patterns

**FIGURE 1:**
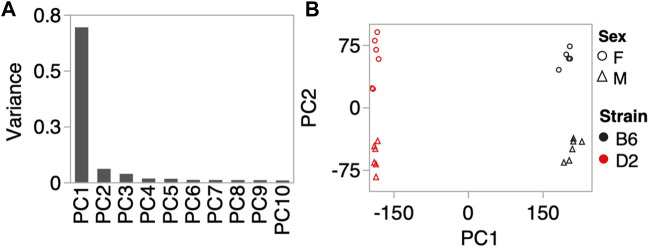
Methylome-wide principal component analysis (PCA). **(A)** PCA was done for the 210,191 CpG regions, and the plot shows the variance explained by the top 10 PCs. **(B)** Scatter plot between PC1 and PC2 shows no batch effect, but samples separate by strain along PC1, and by sex along PC2. We did not detect a methylome-wide effect of CORT+DFP treatment.

To investigate whether the CORT + DFP treatment induces strain dependent changes in DNA methylation, we used MBD-seq to assay the methylome in 23 male and female B6 and D2 mice (Table 1). First, to get a large-scale view of the methylome and to identify the main sources of variance, we applied PCA to the full set of 210,191 CpG regions. The top principal component (PC1) explained 70% of the variance, followed by 6% and 4% for PC2 and PC3, respectively (Figure 1A). A Plot of the top two PCs show that the main explanatory variable is strain, and PC1 segregates the samples by strain. This is followed by sex, which is captured by PC2 (Figure 1B). PC3 weakly captured the variance due to age with Pearson r = 0.5 (*p* = 0.01). None of the top 10 PCs differentiated between the two treatment groups, and this indicates that treatment alone did not have a large-scale effect on the methylome.

### Epigenome-wide association study

**FIGURE 2:**
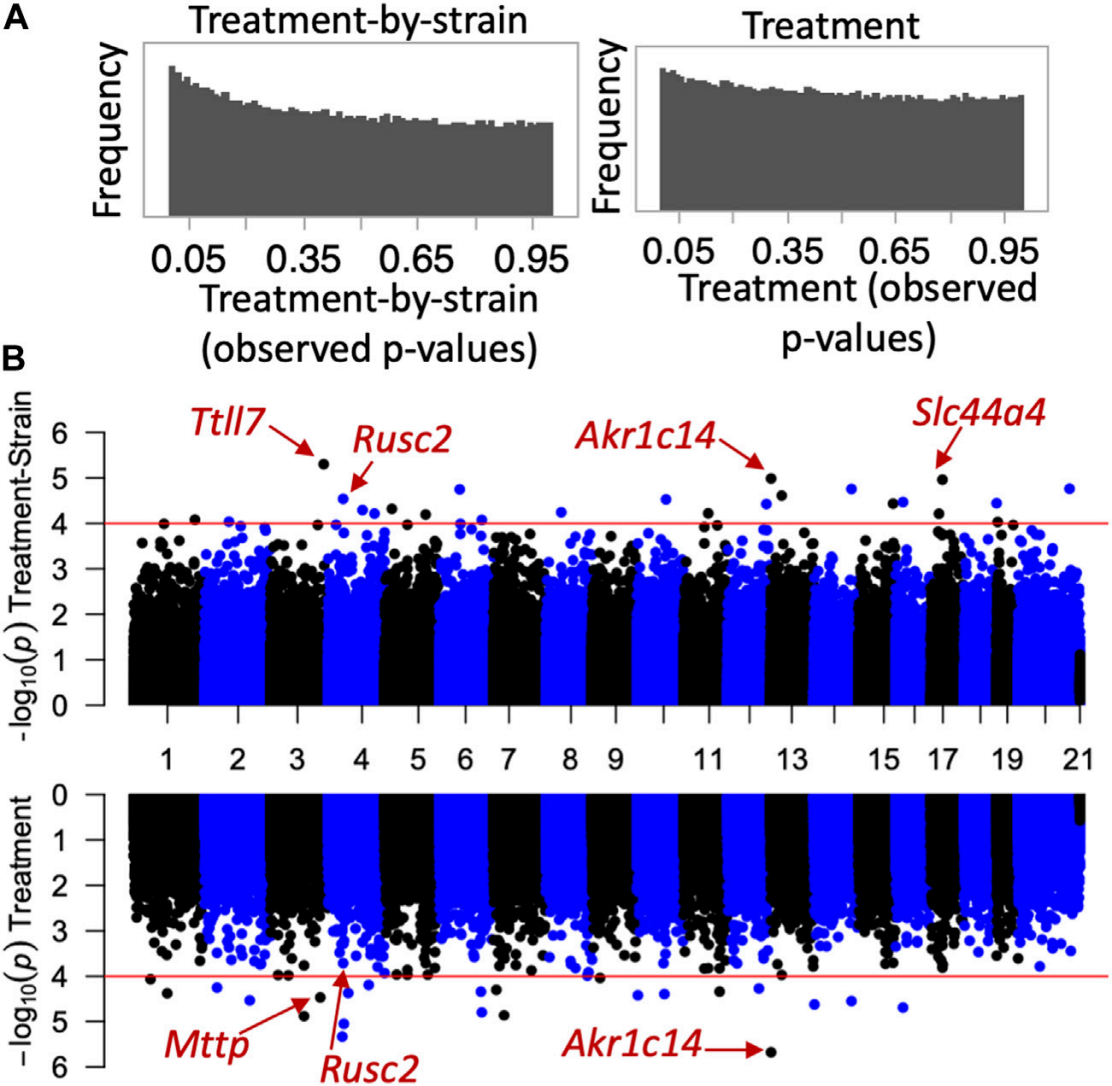
Epigenome-wide association study (EWAS) of CORT+DFP. **(A)** *p*-value histograms from the EWAS show a higher frequency of low *p*-values particularly for the treatment-by-strain interaction effect. This indicates a deviation from the null hypothesis (i.e., a true effect of treatment, but a study that may be underpowered). **(B)** Mirrored Manhattan plots for treatment-by-strain interaction (top), and treatment effect (bottom). The x-axis plots the location of each CpG region (from chromosomes 1 to X), and the y-axis shows the −Log10P. The red horizontal line marks the nominal significance threshold of −Log10P = 4. Locations of few genes of interest are highlighted.

Next, we investigated whether the CORT + DFP treatment results in site-specific changes to the methylome. For this, we applied a multivariate regression model to detect differential methylation (epigenome-wide association study or EWAS) as a function of the main effect of treatment, and the treatment-by-strain interaction with age and sex as covariates. The *p*-value distributions for treatment and treatment-by-strain interaction showed a slight skew towards low *p*-values for interaction effect (Figure 2A). This indicates a true effect of treatment, but a study that is currently underpowered. For this reason, we set a relatively lenient uncorrected statistical threshold of *p* ≤ 0.0001. At this threshold, 36 CpG regions showed a main effect of treatment, and 39 CpG regions showed an effect of treatment-by-strain interaction (Figure 2B). In total, 68 CpG regions in or near 66 genes showed differential methylation at *p* < 0.0001 due to treatment and/or treatment-by-strain interaction (Supplementary Data S1).

**FIGURE 3:**
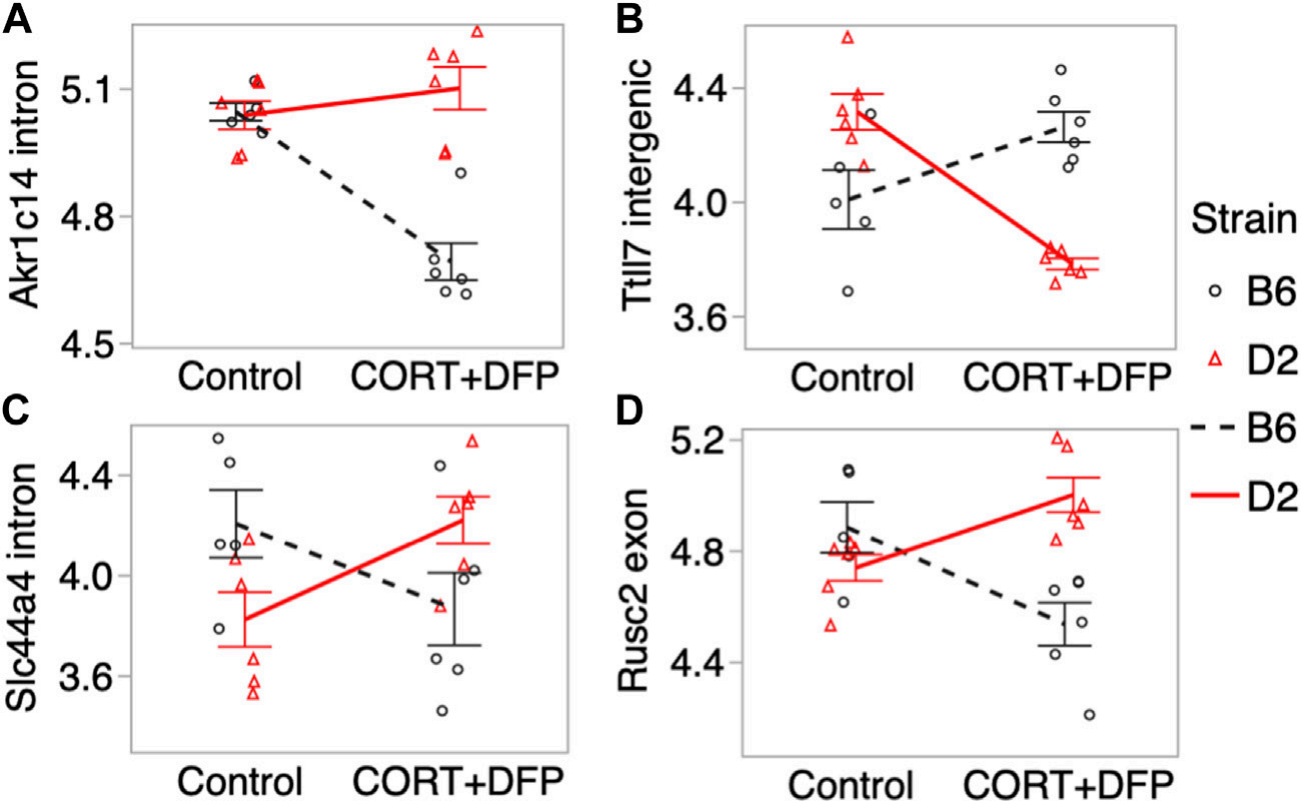
Differential methylation of CpG regions in the prefrontal cortex. Plots show the methylation patterns in control versus CORT+DFP treated C57BL/6J (B6; black), and DBA/2J (D2; red) mice. CpG regions located in **(A)** an intron of *Akr1c14*; **(B)** intergenic region near *Ttll7*; **(C)** intron of *Slc44a4*; and **(D)** exon of *Rusc2*. Error bars are standard error.

For the main effect of treatment, the strongest EWAS hit was for a CpG located in an intron of the aldo-keto reductase gene, *Akr1c14*. This CpG regions also showed a significant treatment-by-strain interaction, and methylation decreased in the B6 mice following the CORT + DFP treatment but remained unchanged in the D2 mice (Figure 3A). Other examples of CpGs with strong treatment-by-strain interactions include intergenic CpGs near the neuronal tubulin glutamylation gene, *Ttll7* (Figure 3B) (Garnham et al., 2015), CpGs located in an intron of the choline transport gene, *Slc44a4* (Figure 3C), and CpGs in the exon of *Rusc2* (Figure 3D).

### Impact on gene expression

**FIGURE 4:**
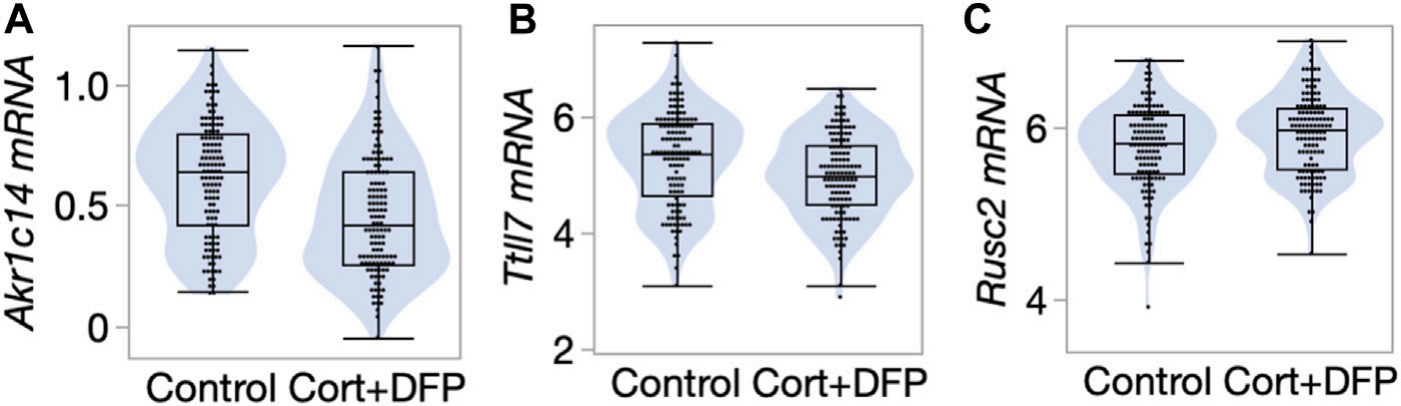
Differential gene expression in the prefrontal cortex. Violin and box plots comparing expression levels of **(A)** *Akr1c14* (mean ± SD of 0.61 ± 0.24 in control; 0.47 ± 0.25 in CORT+DFP; *p* < 0.0001), **(B)** *Ttll7* (5.3 ± 0.83 in control; 4.99 ± 0.72 in CORT+DFP; *p* = 0.004), and **(C)** *Rusc2* (5.78 ± 0.49 in control; 5.93 ± 0.46 in CORT+DFP; *p* = 0.01). 127 control mice, and 129 CORT+DFP treated mice; represents 32 BXD member strains.

We next referred to a previously published RNA-seq based gene expression data from the PFC (Xu et al., 2020). The transcriptomic data was generated from a large cohort of mice belonging to the BXD Family (the progeny of B6 and D2) (Xu et al., 2020) and used the same control versus CORT + DFP treatment. We examined whether the differentially methylated CpGs also showed concordant treatment dependent differential expression. 58 of the 68 CpG regions detected by the EWAS were paired to the corresponding cognate genes (defined as the nearest gene for intergenic CpG regions, and gene in which the CpGs are located for genic regions). At a nominal *p*-value <0.05, 18 transcripts showed differential expression due to treatment (Supplementary Data S1). Of these, the strongest treatment effect was on the expression of *Akr1c14*, and in this case, the CORT + DFP treatment resulted in a strong downregulation in gene expression (Figure 4A). The genes with differential expression also included *Ttll7* (Figure 4B) and *Rusc2* (Figure 4C). Overall, our results highlight a few genes (e.g., *Akr1c14, Ttll7, Rusc2* and few others in Supplementary Data S1) as potential epigenetic targets of CORT + DFP treatment that could also impact gene expression.

## Discussion

The major aim of this work was to demonstrate genetic differences in the epigenetic contribution to individual differences in the chronic nature of Gulf War illness. We follow the work of Ashbrook et al. (2018) who reported histone modification and altered gene expression in the brain following treatment with corticosterone and diisopropylfluorophosphate (DFP), a sarin surrogate, in the C57BL/6J mouse. In the Ashbrook study, the authors reported corticosterone plus DFP to alter expression of genes related to immune and neuron functioning. Here, we report strain-related differential expression of four genes, *Ttll7, Akr1c14, Slc44a4,* and *Rusc2. Ttll7*, tubulin tyrosine ligase-like protein 7, is highly expressed in the brain, including cortical, striatal and limbic areas. It is also highly expressed in the aorta. Its documented function is promotion of neurite growth (Ikegami et al., 2006). *Slc44a4*, sodium-dependent choline transporter is highly expressed in cholinergic neurons in colon, duodenum, gall bladder, kidney and lung. *Rusc2, Run and Sh3 domain containing 2* are highly expressed in the brain, somewhat less so in adrenals, endometrium, heart, kidney, ovaries and lungs. One biochemical function is intracellular protein trafficking. Mutations have been implicated in microcephaly and intellectual deficiency.

**FIGURE 5:**
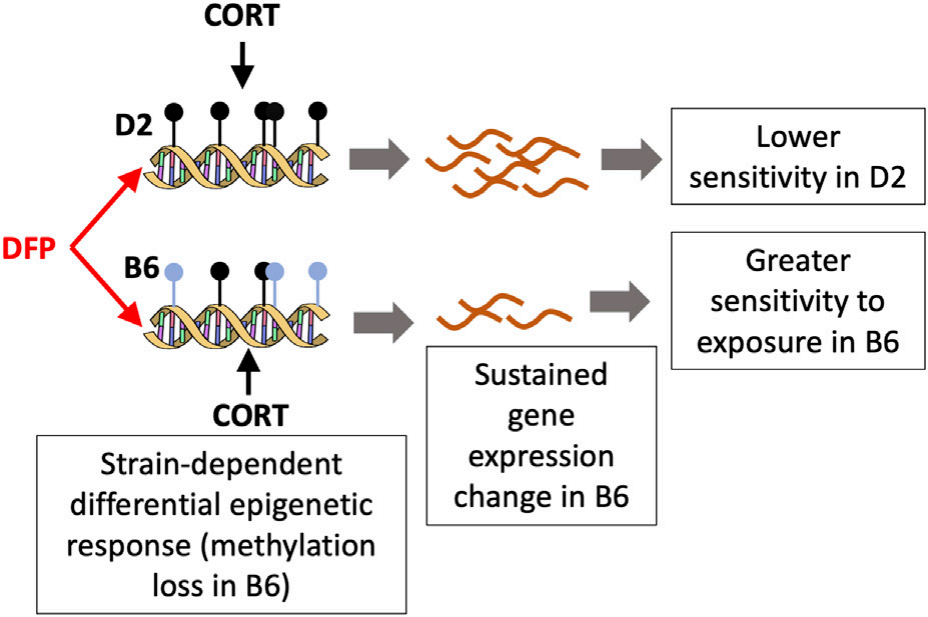
A model for the differential response to toxicant exposure based on the methylation pattern for *Akr1c14*. The C57BL/6J (B6) mice show a loss in methylation following DFP+CORT treatment while the DBA/2J strain remains mostly unchanged. The associated decrease in the expression if *Akr1c14* may result in a sustained and greater sensitivity in B6 compared to D2.

The strongest strain dependent change in DNA methylation that we detected was for the CpG region located in the intron of *Akr1c14, aldo-keto reductase family 1, member C14*. *Akr1c14* is highly expressed in liver, kidney, and colon, and participates in the development of Leydig cells and in developing and mature cells, in the production and release of androgens (Pratt-Hyatt et al., 2013). Particularly relevant to the effects of environmental toxicants, the aldo-keto reductase (AKR) family has a conserved role in drug metabolism and detoxification (Barski et al., 2008). In *Escherichia coli*, the aldo-keto reductase gene, AKR5F1, a homolog of the mammalian AKR family, plays a role in metabolizing organophosphorus compounds found in soil (Jiang et al., 2007). In our present work, we found that while DNA methylation in the intron of the *Akr1c14* gene in D2 remained mostly unchanged following CORT + DFP treatment, there was a significant decrease in methylation in B6 in response to the treatment (see Figure 3A). We note that DNA methylation has a complex relation with gene expression, and while methylation of promoter CpGs generally have an inverse correlation with gene expression, CpG methylation in introns and exons can have a positive correlation with gene expression (Jones, 1999). While we are not able to perform a well-powered test of strain-by-treatment interaction for the expression of *Akr1c14* due to low sample size, we find preliminary evidence that the B6 strain shows a significant downregulation in expression in the PFC following treatment. As this expression analysis in the two strains had only two D2 mice for the treatment group, this figure is presented as a supplemental (Supplementary Figure S1). In the progeny BXD strains, we find a highly significant downregulation in the expression of *Akr1c14* following treatment. Overall, our observations suggest that epigenetic modification in response to an organophosphate such as DFP may mediate some of the sustained and long-term changes that cause symptoms of GWI to persist long after exposure. Based on the B6 by D2 difference seen for the *Akr1c14* gene, we suggest a possible model (Figure 5). The genetic basis for this differential response is yet to be determined, and it may be related to differences in stress response between the two strains (Matthews et al., 2008; Mozhui et al., 2010; Terenina et al., 2019).

Considering the multiple functions of these 4 genes, we are reminded that functional proteins often do much more than that for which they are named. Accordingly, variants in *TTLL7* may be involved in cognitive difficulties seen in chronic GWI, *AKR1C14* may be involved in the chronic gastrointestinal complaints, *SLC44A4* and *RUSC2* would be operators in parasympathetic system-related complaints. This refinement of genes whose expression has been altered more or less permanently may provide clues as how to treat the myriad symptoms by targeting biochemical pathways related to the nominated genes.

## Conclusion

Here, we have proof-of-principle of genetic-based differential epigenetic response to exposures that mimic the exposures that Gulf War veterans experienced. This is important considering that GWI has multiple, seemingly unrelated effects that differ across individuals. Also, because we have this evidence of genetic differences in the epigenetic effects of the exposure in the parental strains of the extant 140 BXD recombinant inbred strains, this supports the charge to expand this work to include 30 or more of the BXD strains and both sexes so that we may conduct genetic mapping to nominate candidate genes underlying the individual differences.

## Data Availability

The datasets presented in this study can be found in online repositories. The names of the repository/repositories and accession number(s) can be found below: https://www.ncbi.nlm.nih.gov/geo/, GSE225179.
