# Supplementary material for: Epigenetic analysis in a murine genetic model of Gulf War illness
Source: Front Toxicol. 2023 Jun 14;5:1162749. doi: 10.3389/ftox.2023.1162749 (PMC10300436; doi:10.3389/ftox.2023.1162749)
Supplement: Supplementary file 1 [file Image1.pdf]

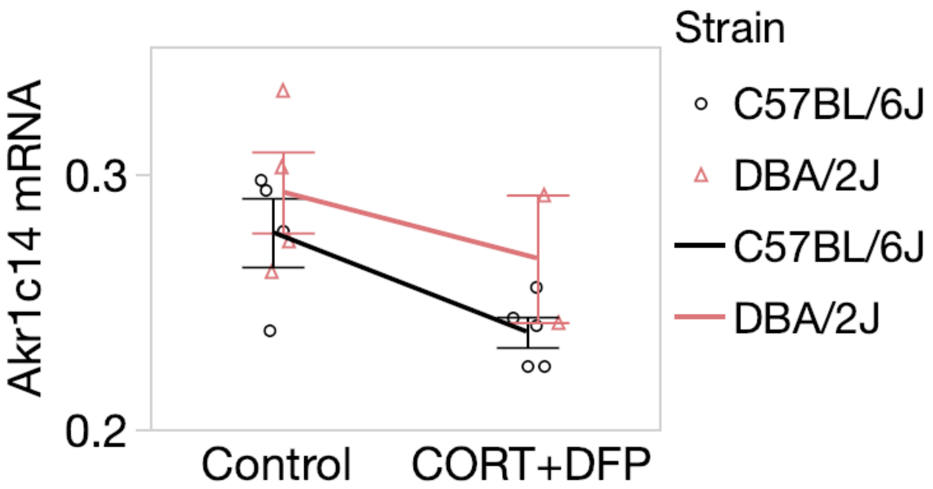

Figure S1. Expression of *Akr1c14* in the prefrontal cortex of C57BL/6J and DBA/2J mice.
Gene expression of *Akr1c14* in the control and treated C57BL/6J (black) and DBA/2J (red) mice. Based on t-test stratified by strain, the CORT+DFP treated C57BL/6J have a significantly lower expression ($p = 0.02$). The DBA/2J mice show no difference (however, note that the data is from only two DBA/2J mice in the treatment group, and this is insufficient sample size for a full regression model that includes an interaction term).
